# Public Interest in Janus Kinase (JAK) Inhibitors for Alopecia Areata: A Google Trend Analysis

**DOI:** 10.2196/75119

**Published:** 2026-04-24

**Authors:** Jade Howard, Nourine A H Kamili, Hala Idris, Loren D Krueger

**Affiliations:** 1 Transitional Year Residency Program and BayCare Health System (Winter Haven), College of Medicine, Florida State University, 1201 1st St S, Suite 100A, Winter Haven, FL, 33880, United States, 1 (863) 280-6085; 2 Department of Dermatology, School of Medicine, Emory University, Atlanta, GA, United States

**Keywords:** alopecia areata, hair loss, Janus kinase inhibitors, Google Trends, alopecia areata treatment, hair loss treatment

## Abstract

Public interest in Janus kinase (JAK) inhibitors for alopecia areata increased following media coverage and the 2022 US Food and Drug Administration (FDA) approval of baricitinib, highlighting the need for patient education and physician guidance on appropriate indications and treatment selection for hair loss disorders.

## Introduction

Janus kinase (JAK) inhibitors are a class of medications that work by targeting and inhibiting JAK enzymes, which play a significant and diverse role in the immune system. The JAK system has been implicated in a variety of immune pathways including inflammation and autoimmunity [1]. On June 13, 2022, the US Food and Drug Administration (FDA) approved the use of a JAK inhibitor, baricitinib, commercially known as Olumiant, for adults with severe alopecia areata, an autoimmune form of hair loss [2]. Additionally, this medication was also approved for the use of severe alopecia areata in children aged 12 years and over on June 26, 2023. The objective of this study was to assess the impact of the FDA approval of the JAK inhibitor, baricitinib (Olumiant), for severe alopecia areata on public interest in JAK inhibitors for alopecia areata.

## Methods

To evaluate public interest, we used Google Trends, a free analysis tool that provides insight into public interest for search terms. Google Trends calculates a relative search volume (RSV), ranging from 0 to 100. An RSV value of 0 indicates minimal interest in a keyword and a value of 100 represents the peak interest within a given period [3]. For our study, we searched the following keywords from January 2014 to January 2024: “JAK inhibitor,” “JAK inhibitor + hair loss,” and “JAK inhibitor + alopecia areata.” These keywords were chosen to encompass generic and specific terms related to the topic of JAK inhibitors and hair loss.

## Results

**Figure 1.**
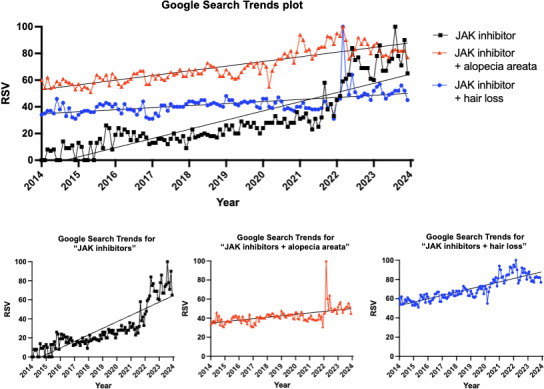
Google Trends results for the search items “JAK inhibitor,” “JAK inhibitor + alopecia areata,” and “JAK inhibitor + hair loss” (data accessed April 30, 2024). Data are presented as relative search volume (RSV), where RSV of 100 represents peak search activity in a time period. The plot represents the time series analysis of Google Trends RSV per month between January 2014 and December 2023 for the terms “JAK inhibitor,” “JAK inhibitor + alopecia areata,” and “JAK inhibitor + hair loss.” Search interest for “JAK inhibitor” increased at a rate of 6.89 (95% CI 6.16‐7.62; *P*<.001; *R*²=0.748) RSV units per month. Searches for “JAK inhibitor + hair loss” and “JAK inhibitor + alopecia areata” increased at a rate of 3.50 (95% CI 3.13‐3.87; *P*<.001; *R*²=0.749) RSV units per month and 1.54 (95% CI 1.12‐1.96; *P*<.001; *R*²=0.306) RSV units per month, respectively. JAK: Janus kinase.

A time series analysis was used to assess changes in RSV between January 2014 and December 2023. Linear regression demonstrated significant upward trends in RSV for all 3 search terms (Figure 1). Search interest for “JAK inhibitor” increased at a rate of 6.89 (95% CI 6.16‐7.62; *P*<.001), RSV units per month with a strong model fit (*R*²=0.748). Searches for “JAK inhibitor+ hair loss” also increased significantly, at a rate of 3.50 (95% CI 3.13‐3.87; *P*<.001; *R*²=0.749) RSV units per month. Similarly, “JAK inhibitor+ alopecia areata” demonstrated a statistically significant upward trend, though with a smaller magnitude of increase (1.54, 95% CI 1.12‐1.96 RSV units per month; *P*<.001) and a more modest model fit (*R*²=0.306).

## Discussion

All 3 search terms (“JAK inhibitor,” “JAK inhibitor + hair loss,” “JAK inhibitor + alopecia areata”) demonstrated significant increases over time (*P*<.001), indicating rising public interest from January 2014 to December 2023. Searches for “JAK inhibitor + hair loss” showed a strong and consistent upward trend (*R*²≈0.75), while general searches for “JAK inhibitor” increased at the fastest rate, suggesting expanding overall awareness of the drug class. The increasing trend is not perfectly linear; rather, it reflects periods of spikes, drops, and plateaus. This variability is possibly influenced by social factors such as periods of media coverage, FDA approvals, etc. For example, there is an appreciable spike in all 3 search terms between 2020‐2023, which may indicate increased public interest following increased media coverage about the FDA approval of JAK inhibitors for severe alopecia reported by news outlets as early as 2019 [4]. Baricitinib became the first FDA-approved JAK inhibitor for alopecia areata in 2022. This was followed by the approval of ritlecitinib in 2023 and deuruxolitinib in 2024 [5]. Additionally, social media applications like TikTok could be possible contributors to the increase in public interest, due to user-friendly content, the ability of content creators to freely express their journey with hair loss and JAK inhibitors, and users being able to engage in conversations with their peers anonymously. Although detailed TikTok data trends from 2014‐2023 were not readily attainable, the TikTok Creator Search Insights tool, which provides trends for the past 6 months, indicates that more than 1 million searches were made on TikTok for JAK inhibitors and alopecia from September 2025 to February 2026. These searches yield a variety of videos, including content from patients describing their experience with JAK inhibitors. A more in-depth exploration of search trends and content on social media platforms would provide further valuable insight into the trends in public interest on this topic.

Given the increased public interest in JAK inhibitor treatment as indicated by Google Search Trends in this study, it is important that the public receives proper education regarding the implications of taking JAK inhibitors and the knowledge that JAK inhibitors are treatment for severe alopecia areata, which is an autoimmune condition, and not currently efficacious for other hair loss disorders. It is important for physicians to educate their patients on hair loss disorder treatments and increase patient awareness of certain hair loss treatments for the patient’s form of hair loss.

## Abbreviations

FDA: US Food and Drug Administration
JAK: Janus kinase
RSV: relative search volume
